# A 56-Day Oral Toxicity Study of the Aqueous Extract of* Rapanea melanophloeos* (L.) Mez in Rats

**DOI:** 10.1155/2016/7403087

**Published:** 2016-12-26

**Authors:** Hesbon Z. Amenya, James M. Mbaria, Andrew G. Thaiyah, Grace N. Thoithi, Peter K. Gathumbi

**Affiliations:** ^1^Department of Public Health, Pharmacology and Toxicology, University of Nairobi, P.O. Box 29053-00625, Nairobi, Kenya; ^2^Department of Clinical Studies, University of Nairobi, P.O. Box 29053-00625, Nairobi, Kenya; ^3^School of Pharmacy, University of Nairobi, P.O. Box 19676-00202, Nairobi, Kenya; ^4^Department of Veterinary Pathology, Microbiology and Parasitology, University of Nairobi, P.O. Box 29053-00625, Nairobi, Kenya

## Abstract

*Rapanea melanophloeos* is a tropical tree that is extensively utilized in African traditional medicine to treat helminthiases, tuberculosis, and heart-water. As with many other medicinal plants, there is insufficient information regarding the safety of therapeutic* R. melanophloeos* extracts. An aqueous extract of* R. melanophloeos* stem bark was administered to Sprague Dawley rats at doses of 100 mg/kg, 300 mg/kg, and 1000 mg/kg for 56 days to characterize its potential toxicity after prolonged dosing. Blood samples were obtained fortnightly for serum chemistry and hematology, while organs were collected at the end of the study. The extract caused an increase in organ weight indices of the kidneys and testis at 300 mg/kg and 1000 mg/kg. Hematological and biochemical examination revealed a drop in leukocyte counts and the hematocrit at 1000 mg/kg dose level, while there was a general but nondose-related elevation in alkaline phosphatase activity. There were time-associated variations in the hematological and clinical chemistry parameters at days 28, 42, and 56 in all dose levels, but most values remained within physiological limits. No pathological lesions were evident at histopathology after treatment with the extract. Our data shows that the aqueous extract of* R. melanophloeos* is not likely to be toxic at the doses tested and provides support to its medicinal use.

## 1. Introduction

Medicinal plants continue to play a crucial role in the provision of healthcare in ethnomedicine and ethnoveterinary medicine [1]. They have been utilized this way since the history of mankind [2, 3]. Given the high cost of mainstream medicine on one hand and easy accessibility of herbal remedies on the other hand, medicinal plants become an attractive healthcare option for less economically endowed populations. Up to 50,000–70,000 plant species, from trees to lichens, have been utilized as medicinal plants [4]. On the contrary, only a small number of these medicinal plant species have been scientifically investigated for safety and efficacy [5]. This fact calls for urgent toxicological examination of widely used medicinal plants and extracts.

*Rapanea melanophloeos *(Myrsinaceae) is extensively utilized as a herbal remedy in traditional medicine in most of southern and eastern Africa. In South Africa, the stem bark is particularly widely used, especially when prepared as a decoction [6]. Among its many therapeutic applications,* R. melanophloeos* is commonly utilized in the treatment of helminthiasis [7, 8], tuberculosis [9, 10], and heart-water [6]. It is also used as an expectorant, emetic, and astringent [10], as an anti-inflammatory and analgesic [11].* R. melanophloeos* contains the benzoquinones embelin and rapanone [12], tannins [10, 13], and saponins [14] as the main biologically active components. Pharmacologically,* R. melanophloeos *possesses anthelmintic activity [15], molluscicidal activity [14, 16], anticoagulation properties [10], and fungicidal activity [14]. Also, anticancer activity has been reported by Fouche et al., the antimalarial activity of the leaf extracts by Clarkson et al., and the antimycobacterial activity by Lall and Meyer, 1999 [17].

Previously, in an acute toxicity study, we found that the aqueous extract of* R. melanophloeos* practically nontoxic even at very high doses [18] and that its chloroformic extract does not induce toxicity related changes in organ structure and hematological and biochemical parameters after subacute oral administration for 28 days [19]. However, there is no toxicological evidence on effects of prolonged administration of the more widely utilized aqueous extract in ethnomedicine and ethnoveterinary medicine. In the current study we sought to analyze the 56 day oral toxicity of* R. melanophloeos* stem bark aqueous extract in laboratory rats. During this period, we monitored the clinical signs and hematological, clinical chemistry parameters of the animals. Our data show that the extract is nontoxic, since no organ pathology resulted from its administration despite the alterations in hematological and biochemical parameters. This study supports the prudent use of* R. melanophloeos* in ethnomedicine and ethnoveterinary medicine at the dose levels tested.

## 2. Methods

### 2.1. Plant Material

The bark of* R. melanophloeos* was harvested in June 2009 from a natural forest in Narok, Kajiado County, Kenya, with the help of an experienced local herbalist at GPS coordinates S 0 55.237, E 35 54.432. Botanical authentication was carried out at the Department of Land Resources Management and Agricultural Technology, University of Nairobi, by the department's taxonomists, and a voucher specimen was preserved (number 27/2009) at the departmental herbarium. The plant name was verified at http://www.theplantlist.org (Accessed April 17, 2016).

The bark was air-dried and ground into a fine powder, which was then extracted using distilled water by boiling 10 g batches in 100 mL water for 5 min. The extract was filtered through cotton gauze and a double layer of Whatman's filter paper number 4 and then centrifuged at 5000 rpm for 5 min. It was then freeze-dried in a Heto Drywinner 3 freeze drier (HetoHolten, Germany) and stored at −20°C. The percentage yield was 12.2% (w/w).

### 2.2. Experimental Animals

Sprague Dawley rats aged 6–8 weeks were obtained from the Central Veterinary Investigation Laboratories (Kabete, Kenya). They were randomly allocated and housed in cages, each containing 5 rats, male and females separately. The animals had free access to commercial rat chow and drinking water and were allowed 7 days for acclimatization prior to commencement of the experiments. While conducting this study, we adhered to the internationally accepted principles for laboratory animal use and care as outlined in the European Community guidelines (EEC Directive of 1986; 86/609/EEC). The animal experiments were reviewed and approved by the University of Nairobi's Board of Postgraduate Studies (Nairobi, Kenya).

### 2.3. Toxicity Study

This toxicological study was carried out following the Organization for Economic Cooperation and Development (OECD) repeated dose 28-day oral toxicity study guideline [20]. To test the time-linked effect of the extract on the test parameters, we prolonged the toxicity testing period to 56 days, within which we obtained blood samples at fortnight intervals. Forty animals were randomly allocated to three treatment groups of 5 animals per sex and to a control group. The plant extract was administered daily to the treatment groups at doses of 100 mg/kg, 300 mg/kg, and 1000 mg/kg body weight based on individual bodyweights at day 0 and adjusted according to weekly weight changes. Controls were administered with the vehicle (physiological saline) daily for 56 days until the day prior to necropsy. The animals were carefully observed for clinical signs of toxicity.

#### 2.3.1. Blood Sampling and Hematological Parameters

Blood was collected from the retro-orbital venous plexus of anaesthetized animals into Greiner® tubes containing heparin, as described by Moore, 2006 [21]. Blood samples were obtained from treatment and control groups before commencement of treatment and then fortnightly till the end of the study. Bleeding did not exceed recommended volumes [22]. Hematological parameters were analyzed using the MS4 Vet® hematology blood counter (Melet Schloesing Laboratories, Cergy-Pontoise Cedex, France). The parameters included total erythrocyte count (RBC), total leukocyte count (WBC), hemoglobin concentration, the hematocrit, mean corpuscular hemoglobin (MCH), mean corpuscular hemoglobin concentration (MCHC), mean corpuscular volume (MCV), and thrombocyte count.

#### 2.3.2. Biochemical Parameters

Heparinized plasma was obtained by centrifuging blood in heparin at 12,000 rpm for 5 min. Parameters analyzed were total protein, albumin, alkaline phosphatase (ALP), alanine aminotransferase (ALT), aspartate aminotransferase (AST), creatine kinase (CPK), creatinine, and blood urea nitrogen (BUN). These were evaluated using kits from DiaSys (Diagnostic Systems International, Germany) and Thermo Scientific (Thermo Electron, Australia and Fisher Diagnostics, USA).

### 2.4. Pathology

At the end of the study, all the experimental animals were euthanized and their carcasses subjected to general examination and necropsy. Pertinent gross pathologic observations in organ systems were recorded. The liver, kidney, heart, spleen, adrenals, lungs, testicles, and ovaries were weighed before fixation in 10% neutral buffered formalin.

Standard histopathological procedures were applied [23]. Formalin fixed tissues were trimmed at 2-3 mm thickness, embedded in paraffin, sectioned at 4-5 um thickness, and stained with haematoxylin and eosin (H&E). Microscopic examination was done on the major organs and any organs that showed gross pathology, in all animals at the highest dose group. The findings were compared to parallel sections at lower dosage levels and from control animals.

### 2.5. Statistical Analysis

Data is presented as means and standard deviations for each parameter. Organ weight indices and weight changes were compared by one-way ANOVA, while biochemistry and hematology values were analyzed by two-way ANOVA using Stat View® (SAS Institute Inc., California, USA). The means contributing to variation were outlined using Fisher's least significant difference post hoc test. Differences were statistically significant if *p* < 0.05.

## 3. Results

### 3.1. Clinical Observations

**Figure 1:**
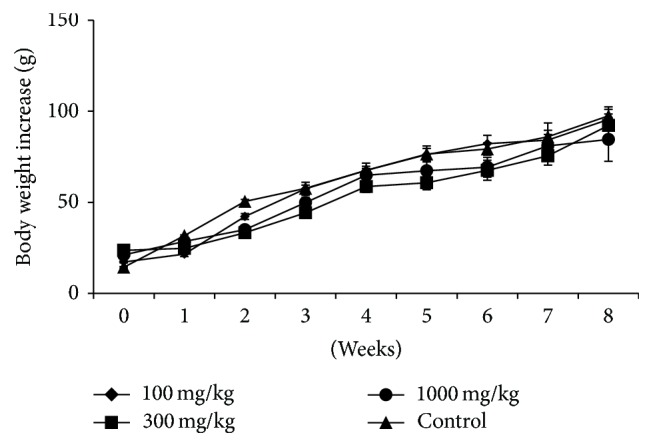
Body weight changes in rats dosed with the aqueous extract of* Rapanea melanophloeos* stem bark for 56 days. There was progressive weight gain in both control and treatment groups.

Dose levels of 100–1000 mg/kg body weight of the extract did not cause any signs of toxicity or deaths in the 8 weeks of treatment. The treatment groups gained weight progressively as in the controls (Figure 1).

### 3.2. Pathological Changes

**Figure 2:**
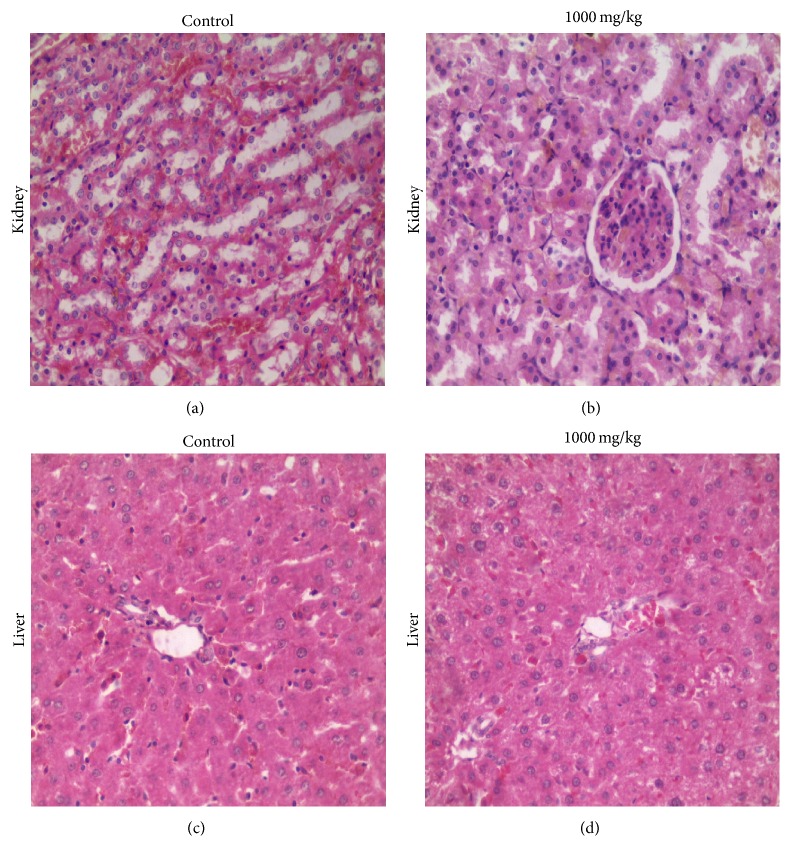
Representative photomicrographs of rats dosed with 1000 mg/kg* R. melanophloeos* aqueous extract for 56 days. (a) The kidney of a control rat showing normal histoarchitecture, (b) the kidney of a treated rat with preserved tubular and glomerular architecture, (c) the liver of a control rat displaying normal histoarchitecture, and (d) the liver of a treated rat showing normal microscopic appearance. H&E ×400.

Organ weight indices (OWI) were calculated as the ratio of the individual organ weight to the carcass weight at necropsy. The OWI of the kidneys at dosage level 1000 mg/kg (*p* = 0.0080) and 300 mg/kg (*p* = 0.0327) were significantly increased compared to the control animals. The testes at dosage level 1000 mg/kg (*p* = 0.0150) and 300 mg/kg (*p* = 0.0016) were also significantly heavier (Table 1). However, there were no significant gross or histopathological changes in any of the organs (Figure 2).

### 3.3. Hematology

**Figure 3:**
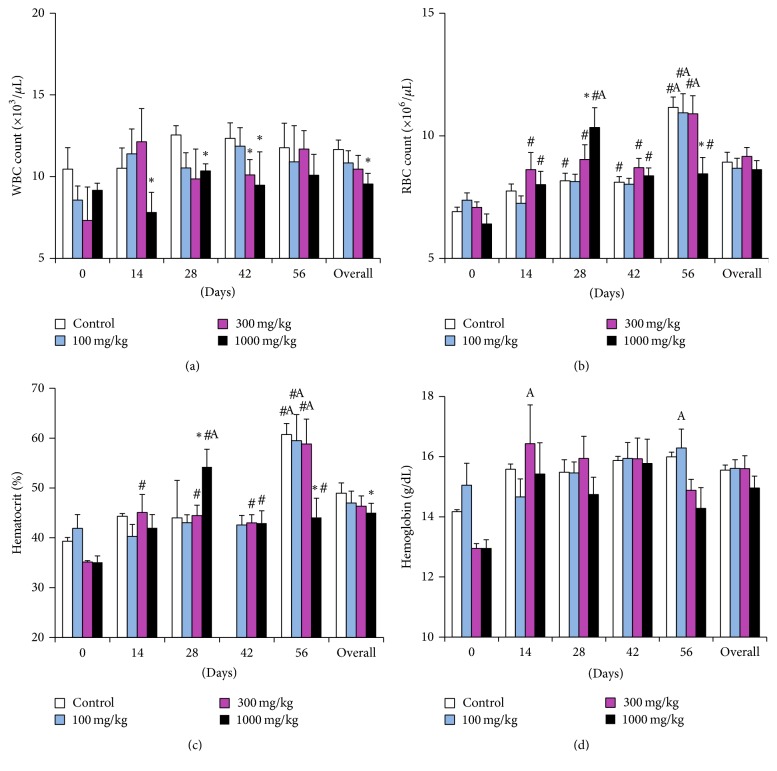
Hematological parameters of rats dosed with the aqueous extract of* Rapanea melanophloeos* stem bark for 56 days. Samples were obtained and analyzed fortnightly; (a) leukocyte count, (b) red blood cell count, (c) hematocrit, and (d) hemoglobin. Overall values are the summary of readings at all time points. *∗* indicates significant difference from the control means (*p* < 0.05, two-way ANOVA followed by Fisher's PLSD post hoc test); # indicates significant difference from pretreatment readings (*p* < 0.05) ^A^values are different from the reference ranges [24].

**Figure 4:**
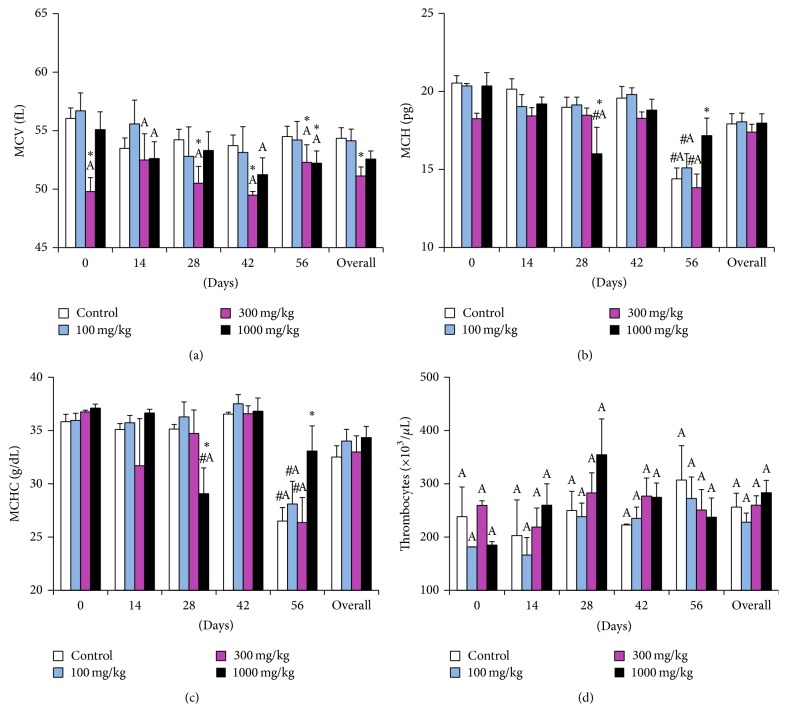
Hematological parameters of rats dosed with the aqueous extract of* Rapanea melanophloeos* stem bark for 56 days. Samples were obtained and analyzed fortnightly; (a) mean corpuscular volume, (b) mean corpuscular hemoglobin, (c) mean corpuscular hemoglobin concentration, and (d) thrombocyte count. Overall values are the summary of readings at all time points. *∗* indicates significant difference from the control means (*p* < 0.05, two-way ANOVA followed by Fisher's PLSD post hoc test); # indicates significant difference from pretreatment readings (*p* < 0.05) ^A^values are different from the reference ranges [24].

There were a significant drop in WBC counts (*p* = 0.0246) and the hematocrit (*p* = 0.0348) at 1000 mg/kg dose level (Figures 3(a) and 3(c)) and MCV values at 300 mg/kg dose level (*p* = 0.0043) (Figure 4(a)) when compared to the controls, but the values were within the physiological limits for rats at all dosage levels. The RBC, hemoglobin, MCH, MCHC, and thrombocytes were not significantly altered by any of the doses but time-linked variations occurred within the experimental period. In comparison to the controls, leukocyte counts of the 1000 mg/kg dose group had an overall decrease from day 14 to day 56, while values of the 100 mg/kg and 300 mg/kg group remained higher (Figure 3(a)). The RBC count increased significantly in all the groups from day 14 up to day 28, notably at the 1000 mg/kg dose level. The most remarkable increase occurred at day 56 in the other dose groups (Figure 3(b)). This trend in RBC count was replicated by that of the hematocrit (Figure 3(c)). Similarly, the hemoglobin concentration increased significantly (*p* = 0.0043), being higher than initial readings at days 14, 28, and 56 with variations between the dose groups (Figure 3(d)). On the other hand, the MCV took a steady decline towards day 56, and values of the 300 mg/kg dose level remained lower than reference values [24] within the experimental period (Figure 4(a)). The MCH readings maintained a stable course up to day 42 after which they dropped sharply towards day 56 in all dose groups. Notably, the 1000 mg/kg dose group had a significant drop at day 28, but values remained higher at day 56 (Figure 4(b)). The MCHC trend replicated that of the MCH (Figure 4(c)). Thrombocyte counts exhibited dose related increases at dosage levels 300 mg/kg and 1000 mg/kg from day 14 to day 28. This trend was lost on days 42 and 56. Overall, only the 100 mg/kg dose group showed a steady increase (Figure 4(d)). All thrombocyte counts remained lower than the physiological ranges [24]. This was also true for RBC and hematocrit and MCH and MCHC at day 56 that were higher and lower, respectively. It was observed that fluctuation in hematology parameters was not dose related over the entire experimental period, but considering the overall effect of both dose and time, the two parameters that showed a dose relationship were the WBC (Figure 3(a)) and the hematocrit (Figure 3(c)), being significantly lower at dose 1,000 mg/kg than those of control group and decreasing step wise with dose. However, the overall effect of dose and time for both values were well within the physiological limits for the laboratory rat.

### 3.4. Clinical Chemistry

**Figure 5:**
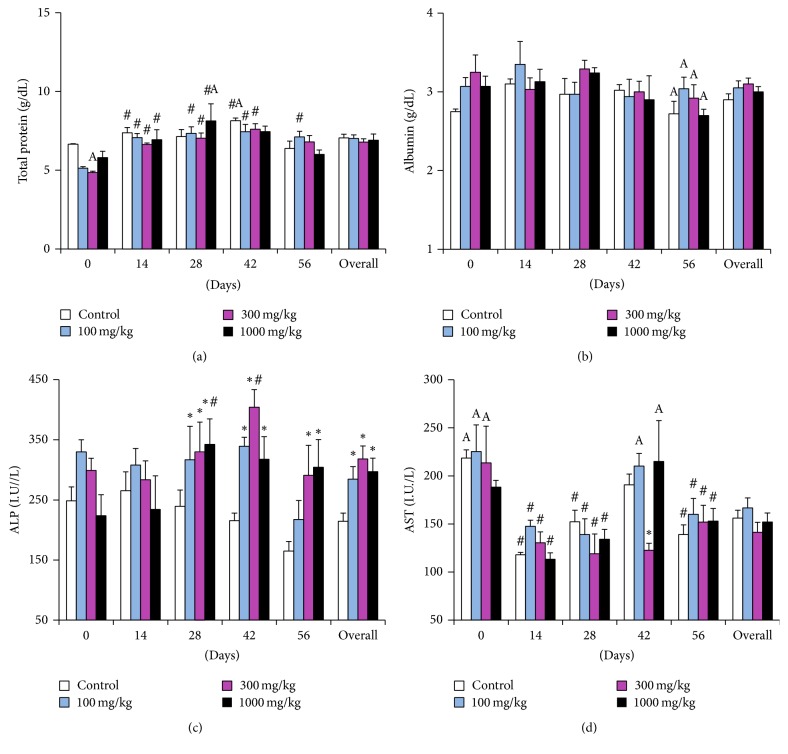
Clinical chemistry parameters of rats dosed with the aqueous extract of* Rapanea melanophloeos* stem bark for 56 days. Samples were obtained and analyzed fortnightly; (a) total protein, (b) albumin, (c) alkaline phosphatase activity, and (d) aspartate aminotransferase activity. Overall values are the summary of readings at all time points. *∗* indicates significant difference from the control means (*p* < 0.05, two-way ANOVA followed by Fisher's PLSD post hoc test); # indicates significant difference from pretreatment readings (*p* < 0.05). ^A^Values are different from reference ranges [25].

**Figure 6:**
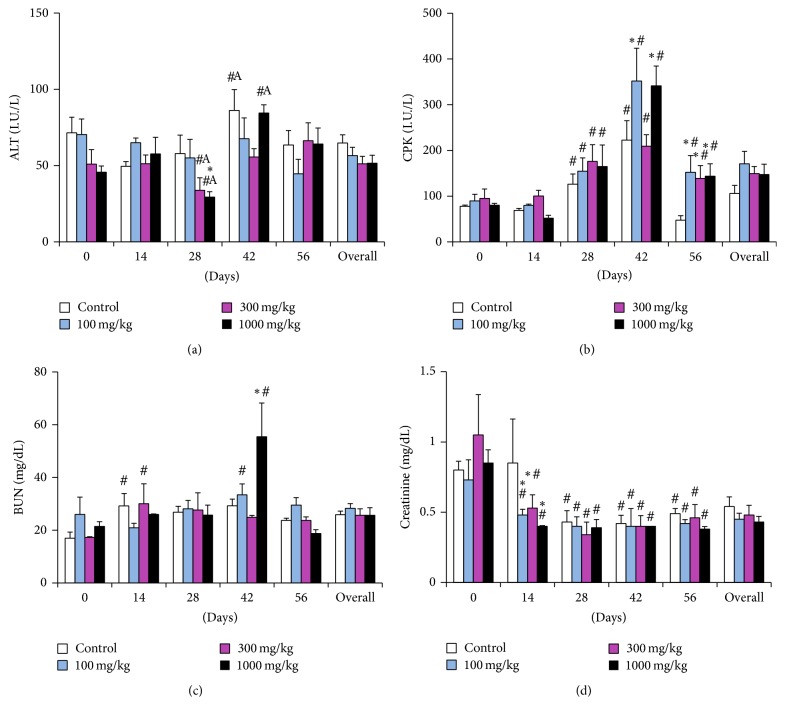
Clinical chemistry parameters of rats dosed with the aqueous extract of* Rapanea melanophloeos* stem bark for 56 days. Samples were obtained and analyzed fortnightly; (a) alanine aminotransferase activity, (b) creatine kinase activity, (c) blood urea nitrogen, and (d) creatinine. Overall values are the summary of readings at all time points. *∗* indicates significant difference from the control means (*p* < 0.05, two-way ANOVA followed by Fisher's PLSD post hoc test); # indicates significant difference from pretreatment readings (*p* < 0.05). ^A^Values are different from reference ranges [25].

Considering the cumulative overall effect of dose and time, there was a significant elevation in ALP (*p* = 0.0034) activity at all the dose levels when compared to the controls, but this elevation did not show a dose related pattern since the overall effect at dose 300 mg/kg was slightly higher than at 1,000 mg/kg. Levels of ALP were within the physiological limit, the overall effect of dose notwithstanding (Figure 5(c)). Also, there was a noticeable nondose-related fluctuation in CPK activity peaking at day 42 (as did other enzymatic parameters) and then declining to levels slightly above the pretreatment values but within the physiological limit (Figure 6(b)). The rest of the biochemical parameters remained unaltered but showed time-related variations within the experimental period.

In comparison with the pretreatment readings, total protein increased significantly (*p* < 0.0001) on day 14, day 28, day 42, and day 56. The trends showed a steady increase in total protein at all dose levels up to day 42, upon which they declined to day 56 (Figure 5(a)). Albumin levels fluctuated around 3 g/dL at all dosage levels and controls. On day 56, however, they showed a slight but significant decline from the pretreatment values in all dose levels and the control group (*p* = 0.0148) (Figure 5(b)). The AST activity declined significantly (*p* < 0.0001) at day 14, day 28, and day 56. There was a modest increment at day 42 in the controls, 100 mg/kg and 1000 mg/kg dose levels (Figure 5(d)). Values on day 0 and day 42 surpassed the physiological range [25].

The ALT activity had nondose-dependent increases at day 42 in the controls and the highest dose level. At day 28, however, there were significant decreases in the 300 mg/kg and 1000 mg/kg dose groups (Figure 6(a)). The BUN levels fluctuated closely to the control values and maintained a steady trend across the experimental period but peaked (*p* = 0.0003) at day 42, particularly in the highest dose group and then declined at day 56 (Figure 6(c)). Creatinine decreased steadily and significantly (*p* < 0.0001) from day 14 onwards to day 56 in all dose levels and the control group (Figure 6(d)). Taking the overall effect of dose in the entire experiment, the biochemical parameters fluctuated around the control, pretreatment values, or physiological limits [25].

## 4. Discussion

Safety evaluation and pharmacological information on medicinal plants is essential to validate their therapeutic application in traditional medicine and to enable informed bioprospecting. In a previous study, we investigated the acute toxic effects of high doses of* R. melanophloeos* aqueous extract in brine shrimps and rats [18]. This study was followed by a subacute oral toxicity study of the chloroformic extract [19]. In both studies the extracts of* R. melanophloeos* were found to be nontoxic. Similarly, our current study provides additional evidence of the safety of* R. melanophloeos* stem bark aqueous extract after prolonged repeated dosing up to 1,000 mg/kg bodyweight for 8 weeks.

In the current study, the aqueous extract did not alter the weight gain or cause adverse clinical signs when compared to the controls. This signifies that the aqueous extract at these doses does not adversely affect the growth rate/body weight gain and corroborates several studies of other medicinal plants confirming their safety [26, 27]. We observed that the relative organ weights of the kidneys and testis were significantly increased at 300 and 1000 mg/kg dosage levels. However, hypertrophy was not evident at histopathology. Further, an analysis of biochemical parameters did not reveal renal insufficiency or soft tissue injury. We therefore concluded that this increase may be of no toxicological significance.

One strength of our study was the fortnight evaluation of hematological and clinical chemistry parameters, to generate a time-course profile of the effect of the extract on these parameters. On hematological examination we observed a decrease in leukocyte counts of the highest dosage level (1,000 mg/kg). On the other hand, since the counts remained within normal ranges [24] and above the pretreatment levels, this extract may have had a mild effect on lymphopoiesis and granulopoiesis. Fluctuation of leukocytes is also associated with progression in age [25, 28]. The RBC counts were not affected at any of the doses, except for a significant increase in the RBC count from day 14 to day 56 in all the dose levels. Consequently, the hematocrit and the hemoglobin also increased from day 14 onwards. We could only attribute this observation to relative polycythemia resulting from changes in body fluid dynamics during in vivo experimental toxicity evaluation [29]. Additionally, Petterino and Argentino-Storino report that hematocrit generally increases in rats with progression of age. The observed increase in hematocrit and RBC values was corroborated by other erythrocytic indices, namely, MCH and MCHC which correspondingly decreased at similar time points alongside relatively stable hemoglobin values. The MCH and MCHC are calculated from hemoglobin, RBC counts, and the hematocrit, respectively. An increase in the RBC or hematocrit will directly reflect in a decrease of the MCH and MCHC, respectively [30]. These trends were observed in our previous subacute toxicity study of the chloroformic extract [19] and are not likely to have arisen from the toxicity of the extract. The MCV is an index of the volume of RBCs which decreases in microcytic anemia or increases in macrocytic anemia [31]. The MCV decline in the 300 mg/kg group was not dose related since the effect was not compounded at the next higher dose group. It may have resulted from interanimal variations and individual animal idiosyncrasies. Thrombocyte counts in the entire study fell below the reference ranges. There is evidence that heparin can cause platelet aggregation [32]. This could explain the low thrombocyte counts. On the other hand, Long Evans rats have been reported to have thrombocyte counts of up to 121–460 × 10^3^/*μ*L [24]. We therefore propose that the extract did not alter thrombopoiesis or platelet function.

An analysis of changes in clinical chemistry parameters within prolonged toxicity studies gives an insight into the mechanism of toxicity of a chemical and the target organ of toxicity [33]. In this study, ALP activity showed a nondose-related increase. The significant elevation of ALP plasma activity in the treatment groups may have been caused by embelin, the main bioactive principle in* R. melanophloeos*. In an earlier study, embelin caused a significant elevation in liver ALP after 6 weeks of administration [34]. We also observed a slight increase in ALP after dosing rats with the chloroformic extract [19], but in both studies, the values did not exceed the physiological limits and they were not dose related.

Overall, in comparison with pretreatment readings, we observed an increase in the total protein, ALP, AST, ALT, and CPK on day 42. These increases occurred concurrently in all the dose levels and the controls and were concomitant with some hematological alterations. We could only attribute this phenomenon to extraneous factors and biological variations resulting from inter- and intraanimal components. Collectively, fluid balance alterations may account for the increase in protein concentration as well as the previous increases in RBC and hematocrit. The increase in liver enzymes may have been caused by a perturbation of liver function. However, this alteration was not severe since most of the values were within physiological limits. These findings, taken together with the absence of histopathological alterations in the liver, show that* R. melanophloeos* may not be hepatotoxic. Other physiological variables such as age, sex, diet, restraint, and circadian effects may have contributed to the overall variation. Creatinine and BUN levels remained unchanged and together with the significant progressive decrease in creatinine from day 0 to day 56 indicate that the extract is nontoxic to the kidney. A significant elevation in the BUN occurred at day 42, probably due to the prevailing alterations in liver function during this period, since BUN concentration is dependent on protein intake and turnover that relies on liver function.

In our earlier acute toxicity study we found the aqueous extract to be practically nontoxic [19]. Furthermore, data obtained from a subacute toxicity study of the chloroformic extract of* R. melanophloeos *has shown that it is not toxic [19]. Also, short-term toxicity evaluation of plants in the Myrsinaceae family showed that they were practically nontoxic to rats [35]. We attribute the low toxicity of* R. melanophloeos* to the innocuous nature of its main bioactive phytocomponents, embelin, and rapanone [36]. The results of this study confirm the fact that there are no reports of any adverse effects due to administration of* R. melanophloeos* water extracts in traditional medicine.

## 5. Conclusions

Overall, the hematology results indicate that the aqueous extract on prolonged administration may cause a slight decrease in leukocytes levels but could harbor no significant toxicological effect, because most of the hematologic parameters remained within the physiological ranges. The absence of histological alteration in organs affirms that prolonged administration of the water extract of* R. melanophloeos* at 100 mg/kg, 300 mg/kg, and 1000 mg/kg will not adversely affect the function of body organs including the liver, kidney, and muscles. The aqueous extract of this plant can therefore be utilized in traditional medicine at oral dosages up to 1,000 mg/kg bodyweight, without posing a risk of prolonged toxicity to the patients.

## Figures and Tables

**Table 1:** Effect of the aqueous extract of *Rapanea melanophloeos* on organ weight indices of rats dosed for 56 days.

Organ	Control	100 mg/kg	300 mg/kg	1000 mg/kg
Liver	4.32 ± 0.54	4.78 ± 0.38	4.31 ± 0.46	4.63 ± 0.34
Spleen	0.46 ± 0.10	0.48 ± 0.14	0.42 ± 0.12	0.36 ± 0.10
Kidney	0.72 ± 0.07	0.85 ± 0.13	0.88 ± 0.22^a^	0.92 ± 0.13^a^
Heart	0.37 ± 0.03	0.41 ± 0.06	0.44 ± 0.09	0.45 ± 0.10
Thymus	0.13 ± 0.04	0.14 ± 0.06	0.14 ± 0.04	0.09 ± 0.03
Adrenals	0.03 ± 0.01	0.04 ± 0.01	0.04 ± 0.01	0.04 ± 0.01
Testes	0.93 ± 0.16	1.17 ± 0.28	1.45 ± 0.18^a^	1.33 ± 0.04^a^
Ovaries	0.05 ± 0.01	0.07 ± 0.02	0.03 ± 0.02	0.04 ± 0.02

The values are expressed as mean ± standard deviation (*n* = 5 animals/sex/dose). ^a^Significant difference from control group (*p* < 0.05, one-way ANOVA followed by Fisher's PLSD post hoc test); organ weight index = (actual organ weight/final body weight) × 100.
